# A Scalable Predictive Modeling Approach to Duplicate Detection in Pharmacovigilance: Algorithm Development and Validation Study

**DOI:** 10.2196/95282

**Published:** 2026-10-09

**Authors:** Jim W Barrett, Nils Erlanson, Joana Félix China, G Niklas Norén

**Affiliations:** 1 Uppsala Monitoring Centre Uppsala, Uppsala Sweden

**Keywords:** pharmacovigilance, artificial intelligence, record linkage, duplicate detection, machine learning

## Abstract

**Background:**

Adverse event reports referring to the same case but treated as independent can negatively impact statistical analysis and may mislead clinical assessment. Pharmacovigilance relies on large databases of adverse event reports to discover potential new causal associations. Their size necessitates computational methods to identify duplicates at scale. The current state-of-the-art is statistical record linkage which outperforms rule-based approaches. In particular, vigiMatch is in routine use for VigiBase, the World Health Organization global database of adverse event reports, and represents the first statistical duplicate detection approach in pharmacovigilance deployed at scale. Originally developed for both drugs and vaccines, its application to vaccines has been limited due to inconsistent performance across countries.

**Objective:**

This study aims to advance the state-of-the-art for duplicate detection in large-scale pharmacovigilance databases and achieve more consistent performance across adverse event reports from different countries.

**Methods:**

This paper extends vigiMatch from probabilistic record linkage to predictive modeling, refining features for drugs, vaccines, and adverse events using country-specific reporting rates, extracting dates from free text, and training separate support vector machine classifiers for drugs and vaccines. Recall was evaluated using 5 independent reference sets. Precision was assessed by annotating random selections of report pairs classified as duplicates.

**Results:**

Precision for the new method was 92% for vaccines and 54% for drugs, compared with 41% for a previous generation method. Recall ranged from 80% to 85% across test sets for vaccines and from 40% to 86% for drugs, compared with 24%-53% for the comparator method.

**Conclusions:**

Predictive modeling, use of free text, and country-specific features advance state-of-the-art for duplicate detection in pharmacovigilance.

## Introduction

### Background

Pharmacovigilance is the science and activities relating to the detection, assessment, understanding, and prevention of adverse events or any other drug- or vaccine-related problem [1]. Randomized controlled trials are performed prior to regulatory approval to establish the efficacy and basic safety of new drugs and vaccines for human use. However, these trials are usually carried out in tightly controlled settings which may not fully reflect real-world conditions. They are not large enough to detect very rare adverse reactions, often exclude vulnerable patients, and do not always run long enough to capture long-latency events. Therefore, the safety and efficacy of drugs need to be continually reevaluated throughout their lifecycle.

Most countries have systems set up to collect individual case reports of adverse events possibly associated with drugs. These are monitored for information suggestive of causal associations between drugs and adverse events or new aspects of known associations, generally referred to as signals of suspected causality [2], and they remain the main source of postmarketing signals [3-5]. VigiBase, the World Health Organization (WHO) global database of adverse event reports for medicines and vaccines, brings together 44 million reports from member organizations across 160 countries that participate in the WHO Programme for International Drug Monitoring (January 2026) [6].

Individual case report systems have inherent limitations, including the largely voluntary nature of reporting. This can affect both reporting rates and data quality, amplifying the requirement for clinical verification of signals before further action. A significant data quality challenge is the duplication of reports. Duplicates are unlinked reports that describe the same case of an adverse event for a specific patient at a certain time [7]. Duplication may result from a failure to link follow-up reports with earlier reports, different reporters submitting reports for the same case, or the same reporter submitting reports regarding the same case to multiple pharmacovigilance organizations. Replication of the same case across databases of multiple organizations is another source of duplication in VigiBase and other databases collating evidence from multiple organizations [8]. This is particularly problematic for reports derived from scientific publications, which are screened by many pharmacovigilance organizations. Duplication can be an obstacle to analysis of individual case reports, affecting both regulatory authorities and pharmaceutical companies [7,9-14]. Pharmacovigilance organizations may combine expert human review with computational and statistical methods in signal detection and analysis, each of which can be misled by duplication. In statistical signal detection, both false positives and false negatives due to inflated counts may distract from more important case series. In expert review of collections of case reports related to a specific drug or adverse event, duplicates may negatively impact review and lead to incorrect conclusions.

To handle duplicates, they must first be detected and managed, which ideally should occur during the reporting or case processing stages. However, this is not always possible, and research has focused on duplicate detection prior to or in association with analysis [7,9-11,15]. However, reports do not always contain enough information to confidently conclude that a given pair are duplicates. Conversely, very similar reports are not necessarily duplicates [16], especially for vaccines where campaigns targeting homogenous patient groups can lead to many similar reports. These problems are exacerbated in settings where data sharing constraints lead to reports with very limited information.

vigiMatch [9], hereafter referred to as vigiMatch2017, has been in continuous operational use for over a decade in VigiBase, and represents the first statistical duplicate detection approach in pharmacovigilance deployed at scale. A performance evaluation within the IMI PROTECT project found vigiMatch2017 to outperform rule-based methods, identifying duplicates in VigiBase that had not been detected by rule-based methods at national regulatory authorities, and some of those which were detected had been misclassified by human assessors overwhelmed by large numbers of false positives [7]. Its real-world deployment has highlighted opportunities for improvement of the method. Specifically, vigiMatch2017’s use of global reporting patterns in its probabilistic record linkage does not generalize well to reports from countries whose drug or adverse event profiles deviate substantially from the global averages, especially in the context of large public health programs and mass administration campaigns, where patients, adverse events and dates of administration tend to have less variance, leading to high false positive rates for some countries.

In this study, we aim to develop and evaluate a duplicate detection method for large collections of individual case reports with improved precision and recall for drugs and vaccines, with consistent results for adverse event reports from different countries. The method takes pairs of adverse event reports from VigiBase as input and performs a binary classification as to whether the reports are duplicates or not. The performance is evaluated by measuring recall and precision on different datasets. Reporting of the study adheres to the Consolidated Reporting Guidelines for Prognostic and Diagnostic Machine Learning Models (CREMLS) [17] (Section S1 in Multimedia Appendix 1). In this paper, we will refer to the new method as vigiMatch2025.

### Related Work

A recent review of duplicate detection methods in pharmacovigilance databases identified 25 scientific papers, most of which did not detail the methods used or their performance [14]. Earlier publications have noted that many methods implemented in commercial and bespoke software are based on rules of various complexity including exact matching [7,13].

The vigiMatch2017 method for duplicate detection for de-identified reports in VigiBase and a similar method by the US Food and Drug Administration (FDA) for the FDA adverse event reporting system (FAERS) have been described and evaluated in scientific publications [7,9-11,15]. Both use probabilistic record linkage according to the principles outlined by Fellegi and Sunter [18] to detect duplicates even with mismatching details. At the same time, they require sufficient matching information, even if reports are identical. Some reflections on the two methods’ similarities and relative strengths have previously been published [19]. We consider them state-of-the-art for duplicate detection in collections of adverse event reports.

## Methods

### VigiBase

VigiBase is the WHO global database of adverse event reports for medicines and vaccines. It is the largest collection of such reports in the world. For this study, we used a fixed version of VigiBase from January 2, 2023, at which time the database comprised approximately 36 million reports. Adverse event reports include information related to the patient, the adverse events, and the medicinal products, which can be either in structured format or free text. They collate information from the original report and any linked follow-up information into one case. Adverse events are coded using MedDRA (the Medical Dictionary for Regulatory Activities terminology), which is the international medical terminology developed under the auspices of the International Council for Harmonisation of Technical Requirements for Pharmaceuticals for Human Use (ICH) [20]. Drugs are coded using the WHODrug Global international reference drug dictionary for medicinal product information [21] with mappings to the Anatomical Therapeutic Chemical (ATC) classification system. VigiBase stores the adverse event reports following the ICH E2B(R2) specification [22].

We defined vaccine reports as any report involving at least one medicinal product in the J07 ATC class characterized in the report as either “suspected” or “interacting” (as opposed to “concomitant”). We defined a drug report as any report which was not a vaccine report. We defined a pair of reports as a “vaccine pair” if it involved at least one vaccine report. Consequently, the set of all drug pairs and the set of all vaccine pairs are disjoint in our analysis. Pairs containing both a drug report and a vaccine report are considered a vaccine pair, since they typically have more in common with pairs of vaccine reports, and to simplify implementation. Due to their overrepresentation and atypical reporting patterns, reports related to COVID-19 vaccines were excluded, leaving a dataset for this study with approximately 27 million drug reports and 1.8 million vaccine reports.

### Reference Datasets

Obtaining representative sets of duplicates for training and evaluating a duplicate detection method through random sampling is intractable, due to the extremely low prevalence of duplicates among all possible report pairs [23]. For this study, we relied on an approach inspired by active learning to train our model and used a range of different reference sets to evaluate its recall. Evaluating the precision required a random sampling of report pairs from the overall dataset and analysis of the pairs predicted as duplicates, as described in the next section. A summary of all labelled data used in this study is in Table 1. Throughout this study, where labels did not exist, report pairs were labelled as duplicates or nonduplicates following the annotation principles described in Section S2 in Multimedia Appendix 1.

**Table 1:** A summary of the reference sets used for training and evaluating the models in this study.

Sources	Number of drug pairs (duplicates/nonduplicates)	Number of vaccine pairs (duplicates/nonduplicates)	Label provenance	Used for training (%)	Used for validation (%)	Used for testing (%)
UMC^a^	630/498	316/373	Historical labelled data and data annotated by at least one annotator during development of vigiMatch2025	60	30	10
FDA^b^ Gold	131/0	0/0	Manually annotated by at least two annotators	0	50	50
FDA Silver	555/0	0/0	Manually annotated by a single annotator	0	50	50
LAREB	1121/0	104/0	118 Manually annotated, 1107 derived from E2B(R2) field A.1.11.2 with help from regulator	0	50	50
AEMPS^c^	1732/0	38/0	16 Manually annotated, 1754 derived from E2B(R2) field A.1.11.2 with help from regulator	0	50	50
MHRA^d^	7260/0	183/0	All pairs derived from E2B(R2) field A.1.11.2 with help from regulator	0	50	50

^a^UMC: Uppsala Monitoring Centre.

^b^FDA: US Food and Drug Administration.

^c^AEMPS: Agencia Española de Medicamentos y Productos Sanitarios.

^d^MHRA: Medicines and Healthcare products Regulatory Agency.

### Uppsala Monitoring Centre Reference Set

A set of 282 known duplicate pairs has historically been used to train vigiMatch2017, which includes the original 38 duplicate groups from its original publication [9]. Further to these pairs, 1535 report pairs were labelled during the development of vigiMatch2025.

Some of these were found by prospectively applying an existing model to extend the reference set (either vigiMatch2017 or an earlier version of vigiMatch2025). Others were found during preliminary evaluations of the model, through debugging its implementation or through experiments guiding feature development. All pairs were manually examined and annotated by at least one coauthor. These pairs were added cumulatively to the training and validation data as they were obtained.

These data were uniformly randomly split into training, validation, and test sets with a ratio of 60:30:10 respectively. The test set was not accessed until the model was finalized, for the performance evaluations presented in this paper.

### Externally Sourced Reference Data

Four members of the WHO programs for international drug monitoring provided lists of confirmed duplicates from their own databases of adverse event reports. A subset of these could be retrieved in VigiBase and were included as reference sets for validation and evaluation of our method. Since these datasets had been labelled independently of one another, and of the Uppsala Monitoring Centre (UMC) reference set, holding them out as recall sets gives a better indication that the model generalizes beyond its training data.

US FDA Center for Drug Evaluation and Research (CDER) provided 2 sets of confirmed duplicates. FDA_Gold contained duplicates identified within 12 case series (2300 reports total, of which 901 comprise 237 duplicate clusters) in the FAERS, doubly annotated by FDA safety reviewers, with adjudication by a third reviewer where necessary. FDA_Silver contained duplicates identified within 26 case series in FAERS (10,165 reports total, of which 3323 comprise 816 duplicate clusters) by the FDA’s duplicate detection system and validated by a single reviewer. Pairs were generated by taking all possible report pairs from a duplicate cluster. From these, 131 duplicate pairs in FDA_Gold and 555 in FDA_Silver contained reports that could be matched to reports in the version of VigiBase used in this study. A report was linked if the FAERS case number matched the value in at least one of E2B(R2) fields A.1.0.1 (Sender’s safety report unique identifier), A1.10.1 (Regulatory authority’s case report number), or A.1.10.2 (Other sender’s case report number). Linking the provided FAERS case numbers to VigiBase is challenging due to other national databases using similar numeric ID schemes, leading to pairs being linked erroneously. However, since this would lead to conservative estimates of recall, we decided not to manually verify all pairs.

With the assistance of the Spanish national regulatory authority Agencia Española de Medicamentos y Productos Sanitarios (AEMPS), the Lareb Netherlands pharmacovigilance center and the UK Medicines and Healthcare products Regulatory Agency (MHRA), we retrieved approximately 10,000 drug duplicate pairs and 325 vaccine duplicate pairs, either with explicitly supplied lists of confirmed duplicates or through linking reports using the E2B(R2) field A.1.11.2, which contains the case identifier from earlier transmissions [22]. By construction, these reports will have an overrepresentation of the “externally indicated” feature described below, which we account for in our evaluation. Since for these organizations we gained explicit confirmation of the validity of this approach to finding true duplicates, we consider them a reliable dataset for evaluation.

These datasets were not used for training. Pairs identified in the UMC dataset were excluded from these test sets. Overall, 50% of each was used for validation, and 50% was held back for the final evaluations in the test sets.

### Duplicate Detection Method

#### Model Overview

vigiMatch2025 computes numerical features designed to capture the similarity or dissimilarity between two reports and combines these into a composite match score for the pair using linear support vector machine (SVM) predictive models. SVMs were chosen because of their computational efficiency, flexibility, ability to perform well with a moderate amount of training data, the explainability of their predictions, and their greater emphasis on training examples close to the decision boundary. Separate SVM models were trained for drug pairs and vaccine pairs, and separate hit-miss models were trained for the relevant features for both drug and vaccine pairs. Features for the predictive models were identified based on a subset of features from vigiMatch2017 complemented by new or modified features.

The following is a short summary of all features used in vigiMatch2025, with detailed definitions in the following sections.

New features in vigiMatch2025:

1. Externally indicated (binary variable)
2. Date embeddings (cosine similarity between convolved binary vectors)

Features in vigiMatch2025 inherited (*) or modified (†) from the vigiMatch2017 features:

1. Patient sex^*^(hit-miss model for categorical variable)
2. Patient age at onset^*^(hit-miss mixture model for a numerical variable)
3. Reported drugs^†^> and adverse events^†^(aggregated into a single feature through summation)
  Reported drugs (country-specific hit-miss model for binary vector)
  Reported adverse events (country-specific hit-miss model for binary vector)
  Compensation for correlations between drug pairs, adverse event pairs, and drug-adverse event pairs (country-specific hit-miss model correlation compensation)
4. Adverse event onset date^*^(hit-miss mixture model for the earliest adverse event onset date listed on the report)

vigiMatch2017 features not included in vigiMatch2025:

1. Patient initials: excluded because they are no longer available in VigiBase
2. Reporting country: excluded since the hit-miss model gives higher scores for matching on countries with fewer reports, effectively setting a lower threshold for suspected duplicates in those countries. In practice, lower overall rates of reporting don’t imply higher rates of duplication. Inclusion of this feature in vigiMatch2017 may have contributed to relatively high false positive rates for some countries.
3. Outcome: excluded since duplicate reports could be expected to have matching outcome (eg, multiple senders) or nonmatching outcome (eg, unlinked follow-up containing updated outcome information). This led to outcome being a weak predictor in practice.

A simple heuristic is also included in the model to exclude pairs that had overall mismatching demographic information (patient age, sex, onset date, and date embeddings), to avoid duplicate classifications being entirely driven by matches on drugs and adverse events. According to this heuristic, pairs were classified as nonduplicates if they had a negative net contribution from the patient age, patient sex, and date embeddings features collectively.

Additionally, vigiMatch2025 incorporates a “blocking” heuristic where it only ever considers report pairs that match on at least one drug (at the WHODrug active substance level) and include adverse events from at least one shared MedDRA System Organ Class. This is the same blocking scheme that is used in vigiMatch2017.

The SVMs were trained based on positive and negative controls from the UMC reference training dataset. To better reflect the class imbalance between duplicates and nonduplicates, and to ensure a diversity of negative controls amongst training samples, we complemented these negative controls with a random sample of 10^8^ pairs. We retained pairs with at least one shared block, resulting in approximately 2.4 million negative controls for the drug model and 6.2 million negative controls for the vaccine model. Due to the extremely low prevalence of duplicates amongst random pairs, this sample is unlikely to contain more than a few, if any, true duplicates.

#### SVM

SVMs are a class of supervised machine learning models. Fundamentally, they find the widest boundary separating the positive and negative classes, while minimizing the number of misclassified examples from the training set. In contrast to many other supervised learning methods, inference is driven entirely by the training examples closest to the decision boundary (the so-called support vectors).

A weighted combination of these support vectors, together with an additional constant vector, defines a hyperplane which is the decision boundary. The sign of the distance between a new datapoint and this plane decides its classification. We chose to fit the model with a linear kernel, so that the decision function for predicting the label *y_i_* for feature vector 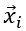
can be written:



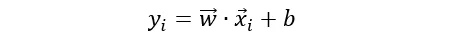



Where 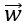 and *b* are the weights and intercept of the fitted SVM.

We used a regularization penalty factor C=1, using the implementation in the sklearn Python library (version 1.3.0) [24], which is in turn a wrapper around the libsvm library [25].

#### Hit-Miss Models

Several of the features in vigiMatch2025 are based on so-called hit-miss models which provide a mechanism to compute the log-likelihood ratio for a specific matching event (eg, both reports are for 5-year-olds, or only one of the reports lists the drug dronedarone), comparing two distinct hypotheses regarding the two reports: (1) they describe the same case; and (2) they describe cases that are independent of one another [26].

With a hit-miss model, matching information is rewarded with greater positive contributions the less frequent the matching values are (eg, two reports matching on a rarely reported drug receive a larger score than two reports matching on a common drug like paracetamol).

Mismatching information is penalized, with greater penalties for features which seldom mismatch on true duplicates (eg, since there is greater coding ambiguity for adverse events than drugs, these are more likely to mismatch, even on true duplicates, and thus mismatching adverse events receive a lower penalty). However, these penalties do not depend on specific values (ie, mismatching on paracetamol carries the same penalty as mismatching on a rarer drug).

The hit-miss model was previously extended to a hit-miss mixture model for numerical features and with a compensation for correlations between binary features [9], which are here applied to patient age and adverse event onset date (hit-miss mixture models) and to drugs/adverse events (correlation compensation).

Both the hit-miss model and the hit-miss mixture model are inherently robust to missing data, inherently yielding zero contributions for any features where either report has missing information.

#### Hit-Miss Models With Country-Specific Drug and Adverse Event Rates

The hit-miss model for drugs and adverse events in vigiMatch2017 uses the overall reporting frequencies of drugs and adverse events in VigiBase to determine the reward/penalty. However, this assumes that these reporting frequencies are globally consistent. In reality, drugs in routine use in high-income countries may not be readily available in lower- or middle-income countries.

For the hit-miss models related to drugs and adverse events, vigiMatch2025 instead considers the reporting frequencies in the country of origin. Where two reports are not from the same country, the global frequencies are used. Parameters of the hit-miss models derived from known duplicates were shared across all countries, due to insufficient training data to infer them per country.

### Date Embeddings

Reports often include multiple dates and a shortcoming of vigiMatch2017 is that it considers only a single date of onset per report and will not reward matches on multiple distinct dates. Of particular interest for duplicate detection are the start and end dates of reported adverse events and drug therapies. Some of these are reported in structured fields, and others are described in the free text narrative of reports. We therefore developed a set of regular expressions to extract and normalize dates from the free text narrative.

In VigiBase, dates are stored as pairs of timestamps reflecting their uncertainty interval (eg, a report of “March 2021” would be represented by 2021-03-01 00:00:00 and 2021-03-31 23:59:59). For our date embedding feature, we included dates which had a maximum uncertainty of 7 days and excluded all dates reported as January 1, regardless of their uncertainty, since this was historically used by some organizations to indicate missing information on the month and day.

We embedded the dates by producing a one-hot-encoded vector for a report, such that each element of the vector represented a single day between 1900-01-01 and 2050-12-31, with the element being 1 for that day if a report includes a date interval starting on exactly that date and 0 otherwise. Adverse event onset date, adverse event end date, drug start date, drug end date, and all dates extracted from the narrative were included in the vector. We applied a convolution with a window size of 7 days to partially reward near matches (Figure 1).

The date vectors of two reports were compared using a cosine similarity score.

**Figure 1:**
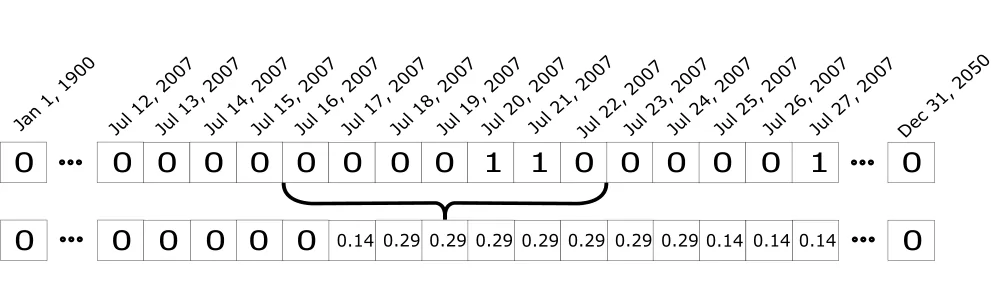
Schematic of how the date vector would be constructed for a report mentioning July 20, 21, and 27, 2007. The top row shows the one hot encoding, and the bottom row the vector after applying a convolution with a window size of 7 days.

### Externally Indicated Reports

As part of the E2B(R2) format, cases can be linked to previous transmissions via field A.1.11.2 [22]. If a report had an ID in field A.1.11.2 (Case Identifier in the Previous Transmission) that exactly matched another report’s value in A.1.0.1, A1.10.1, or A.1.10.2, we defined that pair as “externally indicated.” We encoded this as a binary feature where the value is 1 if a pair is externally indicated, and 0 otherwise. We encoded this information as a feature, rather than using it as a heuristic, since a preliminary investigation estimated its precision in linking reports to be 0.76.

Some IDs are purely numeric, or otherwise generic, which can lead to reports matching erroneously where they coincidentally have the same ID. To mitigate this, we limited matches to IDs starting with one of 30 strings clearly delineating a country (eg, “DE-”, “GB-”, “FR-”, “IT-”. A full list is in Section S3 in Multimedia Appendix 1). With this restriction, approximately 65,000 pairs were linked in this way.

### Benchmark Comparator Method

As the benchmark comparator method for this study, we use vigiMatch2017. It largely follows the description in Norén et al [9], using the hit-miss model and its extensions to compute contributions to an overall match score from each of the following report elements: patient age, patient sex, reporting country, date of onset (for the adverse event), outcome*,* all drugs listed on the report (at WHODrug Active Substance level), all adverse events listed on the report (at MedDRA Preferred Term level), and patient initials. vigiMatch2017 is integrated into the preprocessing of incoming data and can leverage some information not included in VigiBase.

Whereas vigiMatch2025 uses linear SVMs to obtain the overall match score, vigiMatch2017 assumes conditional independence between its features and obtains its total match score through summation of hit-miss model weights (which correspond to log-likelihood ratios). An explicit compensation for correlations between reported adverse events and drugs is included as described above but based on global reporting rates.

The original publication assumed a mixture of normal distributions for the match scores of duplicates and nonduplicates, respectively, to compute a threshold for suspected duplicates [9]. The threshold learned this way was not stable and tended to increase over time. As a pragmatic choice, the vigiMatch2017 implementation was therefore modified to use the mean match score for known duplicate pairs as its threshold for suspected duplicates. A separate heuristic in vigiMatch2017 not included in the original publication, but included in the implementation in routine use, requires that the net contribution from patient age, patient sex, date of onset, and patient initials be greater than 0 for 2 reports to be flagged as suspected duplicates.

Due to high observed false positive rates when applying vigiMatch2017 to vaccine reports during its routine application, it has subsequently only been applied to drug pairs and so can only act as a comparator to the vigiMatch2025 drug model.

### Performance Evaluation

#### Precision Studies

Precision is a measure of how reliable a model’s predictions are. It is defined as:



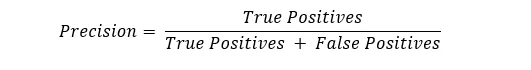



The FDAGold, FDASilver, AEMPS, Lareb and MHRA reference sets contain no nonduplicates, and so they cannot be used to estimate the model’s precision. The UMC reference set contains some labelled nonduplicates, but the biases in how the dataset was built make it unsuitable for measuring precision.

To estimate the precision of the models, a stream of random pairs was presented to each model until it had classified 100 pairs as suspected duplicates. Random pairs were generated from the same sequence of random seeds for each model, in batches of 10^8^ pairs (Note that large groups of duplicates will combinatorially lead to very large numbers of duplicate pairs, meaning they would be overrepresented relative to their report-level prevalence). These predicted duplicates were examined and annotated by a medical doctor (author JFC). In this process, report pairs were labelled as either duplicates, otherwise related, or as nonduplicates according to the annotation guideline (presented in Section S2 in Multimedia Appendix 1). We present precision results both for duplicates and for related pairs (being either duplicates or otherwise related). Author JWB also labelled all pairs to measure interannotator agreement; however, JFC’s annotations were considered authoritative.

These labels were additionally used to estimate the expected number of true duplicates detected per report in the dataset. This was computed as:



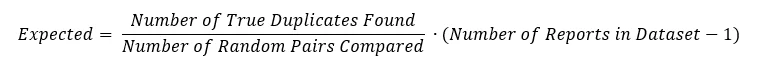



Where the number of reports in the dataset minus 1 is the number of pairs per report. This measure gives an indication of the prevalence of detectable duplicate pairs for each model. Moreover, since vigiMatch2017 and vigiMatch2025 were applied to the same stream of random pairs, a model with a higher rate of detecting true duplicates can be interpreted as having a higher recall.

We also evaluated the performance of the models on report pairs from three individual countries. Two of these were African countries, which are known to have adverse event and drug distributions that are different from the global pattern and had been observed to have high false positive rates by vigiMatch2017. We chose one European country as a comparator, since we expected it to have more comparable reporting to VigiBase globally, due to the large contribution from European countries following the same legislation. The choice of countries was also practical, to ensure there were few enough reports to be able to run exhaustively in all possible pairs. 15 random pairs predicted as duplicates were selected for each country, for each model, and a medical doctor (author JFC) annotated them according to our guideline. We report precision for each country and model. We also computed the number of reports that would remain if only one representative report was kept per *duplicate group.* A duplicate group is defined here as a set of duplicate pairs that are fully connected, so that every report in the group is considered a duplicate of every other report in the group. We find these groups using the networkx Python library version 3.2.1 [27].

#### Recall on Reference Sets

We estimated the models’ recall by applying them to the test reference datasets described in Table 1, and computing recall as:



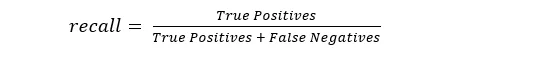



Since some of the reference sets were partially built using E2B(R2) field A.1.11.2, we also report recall after artificially setting the externally indicated feature value to 0, that is, as if no pairs were externally indicated.

## Results

### Trained Models

After training the drug and vaccine models, we investigated the feature importance. Since features were on different scales, the 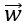 coefficients were not meaningful independently. We thus looked at each feature’s contribution to 
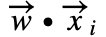
 for known duplicates among the training and validation pairs, and for 1,000,000 random pairs. We compare these to the SVM’s fitted intercept *b* , interpreted here as the evidence threshold.

In Figure 2, we display box plots representing the distribution of contributions from each feature for each model. Pairs excluded by a heuristic are not shown. The distribution for known duplicates is displayed in green, and for the random pairs in purple. For both models, the true duplicates tend to receive higher scores compared to random pairs. The contribution from the drug/AE hit-miss model is typically the largest. Demographic features (ie, age and sex) contribute less to the scores for vaccines than they do for drugs, which is in line with intuition since vaccine recipients tend to be more demographically homogeneous. Mismatches on sex are also much more heavily punished for the vaccine model.

**Figure 2:**
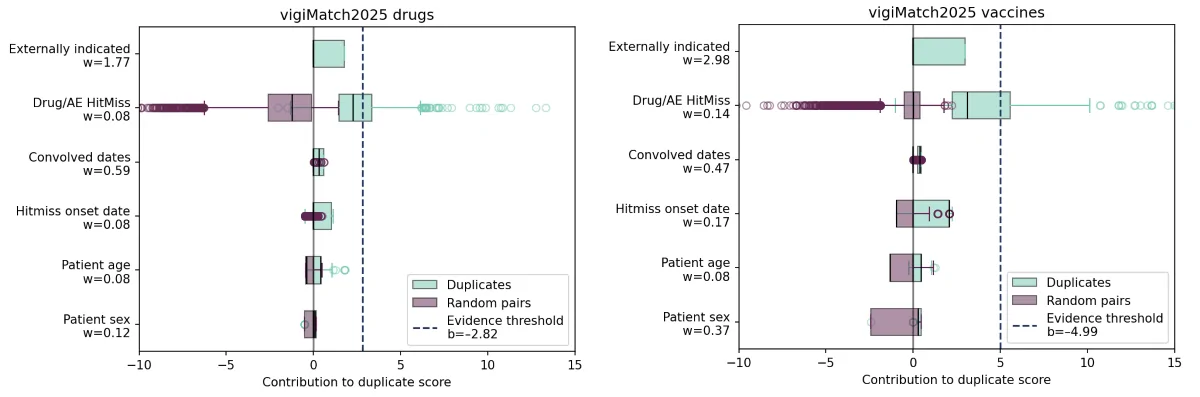
The feature weights and contributions to <inline-graphic xlink:href="ai_v5i1e95282_fig11.png" mimetype="image" xlink:type="simple"/> for true duplicates and random pairs. The fitted SVM weights are given with the y tick labels. Each box plot represents the distribution of the product of the SVM coefficient w and the value of the feature x. Note some extreme outliers for the Drug/AE HitMiss feature are outside the range of these plots.

### Precision Studies

The results for each model are presented in Figure 3 and Table 2. vigiMatch2025 Drugs achieves higher precision than vigiMatch2017, and vigiMatch2025 Vaccines achieves a higher precision still. As seen in Figure 4, the expected number of true duplicates per report is higher for vigiMatch2025 drugs than vigiMatch2017, which indicates a higher recall. vigiMatch2025 Vaccines has a comparable, but slightly lower expected number of true duplicates per report. The interannotator agreement was a Cohen-Kappa score of 0.67, indicating moderate to substantial agreement [28].

**Figure 3:**
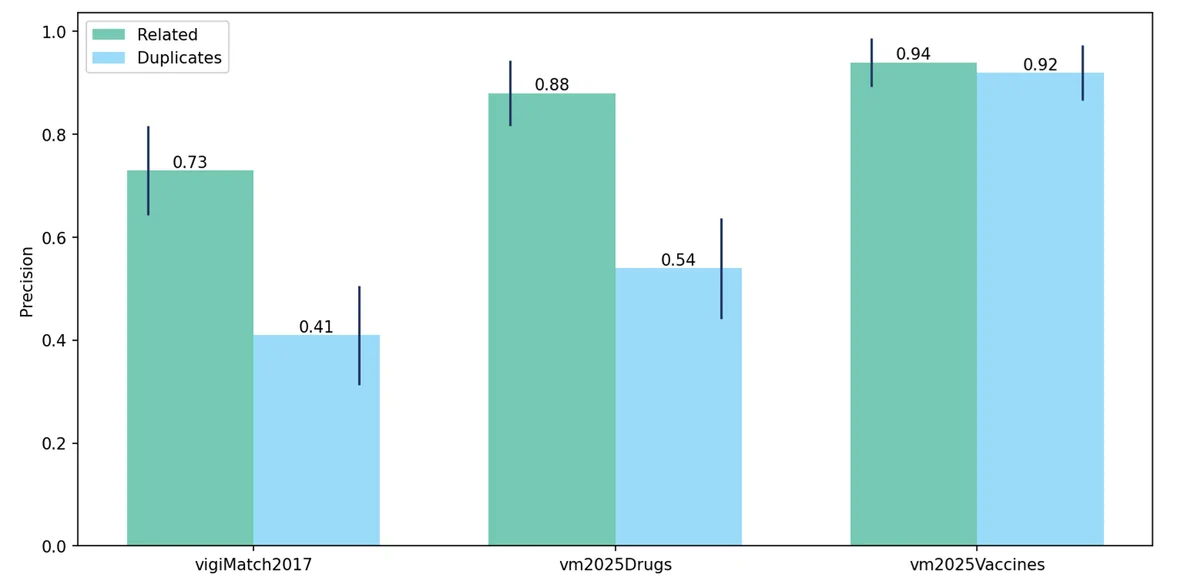
Results from the evaluation of the models’ precision. Vertical lines represent the 95% CI estimated with Wald’s method. The blue bars represent the true duplicates the model is trained to classify. The green bars are reports that are either duplicates or otherwise related.

**Table 2:** Tabulated results from the precision experiments.

Models	Total number of reports from which pairs were drawn (millions)	N random pairs compared (billions)	N predicted duplicates	N true positives	Precision	True positives per billion pairs	True duplicates detected per report
VigiMatch2017	26.9	22.2	100	41	0.41	1.85	0.05
VigiMatch2025 Drugs	26.9	20.5	100	54	0.54	2.63	0.07
VigiMatch2025 Vaccines	1.8	3.2	100	92	0.92	28.75	0.05

**Figure 4:**
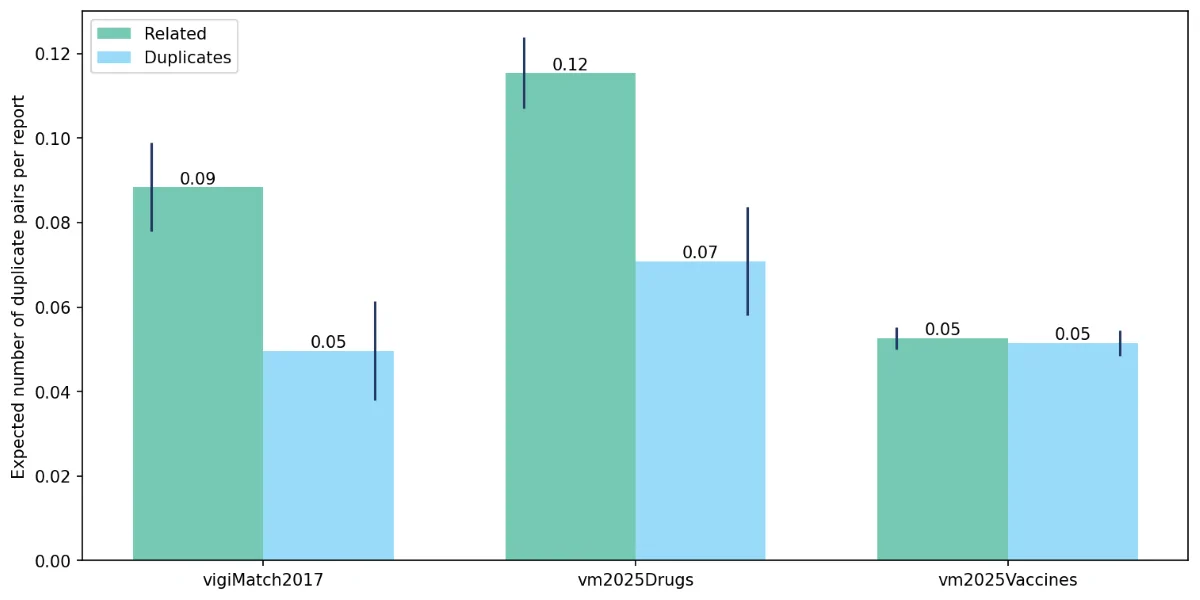
The expected number of true duplicates found per report for each model, with vertical lines representing the 95% CI estimated with Wald’s method. The blue bars represent the true duplicates the model is trained to classify. The green bars are reports that are either duplicates or otherwise related.

Of the 100 pairs predicted as duplicates, one (for drugs) and four (for vaccines) had positive contributions from the externally indicated feature. All these five pairs were true positives and remained as true positives when masking the contribution from the externally indicated feature.

The results for the individual countries are in Table 3. For the 2 African countries, vigiMatch2017 predicts significantly more pairs as duplicates than vigiMatch2025; however, there were no true positives in the two times 15 pairs sampled, whereas vigiMatch2025’s precision was commensurate with the results in Figure 3. The results for vigiMatch2017 and vigiMatch2025 were roughly equal for the European country, with vigiMatch2017 predicting slightly more pairs as duplicates and finding two additional true positives. It is also seen that vigiMatch2025 removes fewer reports via deduplication than vigiMatch2017 for both African countries. While 15 pairs per model, per country are too few to have strong statistical support, the results presented do give a strong indication of a lower false positive rate.

**Table 3:** Results of the individual country experiments, showing each model’s precision on pairs classified as duplicates after exhaustively classifying all pairs in VigiBase from that country.

Countries	African country A	African country B	European country A	
N reports in VigiBase	19,042	4363	1004	
vigiMatch2017 remaining reports after removing suspected duplicates	13,363	3503	976	
vigiMatch2025 remaining reports after removing suspected duplicates	18,756	4243	987	
N possible pairs (millions)	181.3	9.5	0.5	
vigiMatch2017 predicted duplicate pairs	42,933	1969	30	
vigiMatch2025 predicted duplicate pairs	409	137	21	
vigiMatch2017 precision	0/15	0/15	8/15	
vigiMatch2025 precision	6/15	13/15	6/15	

### Recall

Recall for each of the test reference sets is presented in Figure 5. vigiMatch2025 Drugs achieves a higher recall for every dataset, both with and without relying on the externally linked feature. The recall for the vigiMatch2025 Vaccines is comparable to the vigiMatch2025 drug model for most datasets.

**Figure 5:**
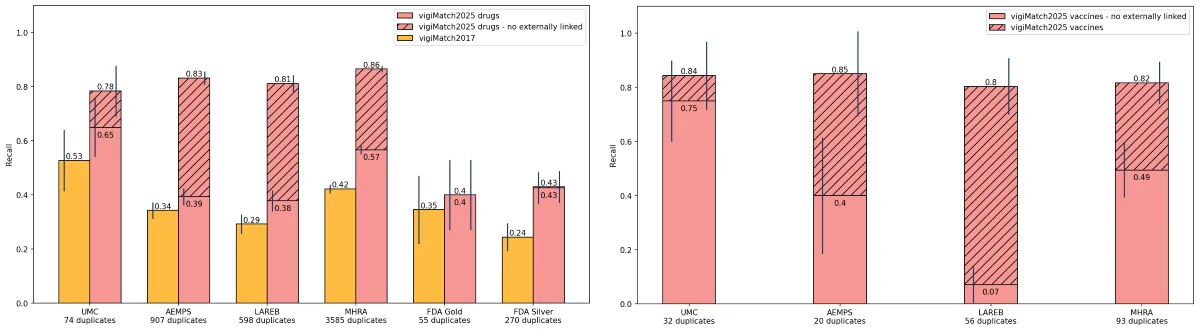
Recall performance of each of vigiMatch2017, vigiMatch2025 Drugs, and vigiMatch2025 Vaccines. The hatched bars represent the complete performance of the model, and the solid bars represent the performance by assuming that no pairs can be linked via the reported duplicate IDs. 95% CIs for the height of each bar are presented in dark blue, computed using Wald’s method.

### Qualitative Review of Cases

A qualitative review was conducted to give a better understanding of where vigiMatch2025 improves upon vigiMatch2017 and where it makes mistakes. A selection of true positives, false positives, false negatives, and true negatives from the test reference set were examined. The examples where vigiMatch2025 succeeded over vigiMatch2017 reflected known limitations of vigiMatch2017, like its inability to account for multiple dates on each report and country-specific reporting patterns. The cases where vigiMatch2025 fails tended to be unusual cases, such as nonduplicates with many matching adverse event terms. The qualitative review is described in detail in Section S4 in Multimedia Appendix 1.

## Discussion

### Principal Findings

vigiMatch2025 demonstrates a substantial improvement in performance over the previous state-of-the-art comparator model vigiMatch2017, while retaining many attractive features of the earlier model. Importantly, the new model achieves improved precision and recall for duplicate detection of both drug and vaccine reports in VigiBase, with a much lower false positive rate for certain countries.

The training set for vigiMatch2025 has been expanded with over 1500 additional labelled pairs. This supports its incorporation of separate SVM predictive models for drugs and vaccines, which allows it to adapt its weights based on the most relevant examples in the training data. This contrasts with vigiMatch2017, which leans heavily on the assumed stochastic model and overall relative frequencies of different reporting elements in the database. However, this increased adaptability comes with an increased risk of overfitting if the training data are selected improperly or later become unrepresentative of duplicates in VigiBase. Another advantage of vigiMatch2025 is that its hit-miss models for drugs and adverse events are trained per country. We saw indications of a significant reduction in the false positive rates in duplicate detection for countries with drug and adverse event distributions that differ from those in the global database. vigiMatch2025 also considers all unique dates related to drugs and adverse events in a report including in the case narrative. This holistic approach to dates allows for a more nuanced comparison of the timelines presented in each report.

At the same time, vigiMatch2025 retains the explainability of vigiMatch2017. The choice of a linear kernel for the SVM makes the prediction a simple weighted sum of the features and a constant intercept, which can be interpreted as an accumulation of evidence and an evidence threshold. The model only classifies a pair as duplicates when there is sufficient evidence. A specific prediction by the model can also be easily interrogated for which elements of the report pair contributed to the model’s prediction, which facilitates end-user interpretation of model output. The evidence threshold can also be adjusted to favor precision or recall, depending on the application.

vigiMatch2025 retains the low computational complexity of vigiMatch2017, which is crucial to most applications. The new features all scale equally well with the number of reports in a database as vigiMatch2017 does, which in the production environment currently compares approximately 1 billion pairs of reports each second on a single Microsoft Azure Standard_D192s_v6 virtual machine (192 vCPUs, 768 GiB RAM). The new representation of dates only requires a dot product of two very sparse vectors at inference time. The country-specific drug and adverse event hit-miss models do not represent any change in computational complexity at inference time over the global model, as they just use different parameters for the same calculations.

The training data used in this study were accumulated during the development of vigiMatch2025, leading to a risk of confirmation bias and overfitting, where the model is specialized to perform well on the specific data it was trained on, potentially hindering its performance on realistic, unseen data. The acquisition of unbiased training data for duplicate detection models poses a significant challenge, due to the extremely low prevalence of positive examples [23], meaning that some level of bias in labelled data is inevitable. As such, the evaluations presented in this paper were constructed to reduce the impact of these biases as much as possible. The precision experiments, by design, give estimates of the models’ performance that reflect real-world prevalence of duplicates. Moreover, several different, independent, and externally sourced datasets, from the FDA, AEMPS, MHRA, and Lareb national pharmacovigilance centers, were used to evaluate the models’ recall, with a fraction of each held out for the final evaluations presented here. However, in future studies, an even more diverse collection of test sets may further probe models’ recall. Moreover, the provenance of the pairs in these datasets, and on what basis they were annotated as duplicates, is not always known.

The annotations themselves also presented a challenge. VigiBase is a highly diverse dataset, and the types of evidence of duplication observed during annotation were equally diverse. Our annotation guideline reflects this diversity of evidence, with room left for expert judgement when reaching a final decision for an annotation. With additional resources, additional manual annotators would also have been desirable for the precision study, although we consider a Cohen-Kappa agreement of 0.67 to be sufficient for the purposes of this study.

National centers often have access to much more information on each report than is included in the anonymized versions shared in VigiBase. Some of the pairs of true duplicates provided for the recall studies do not have sufficient information in VigiBase to be annotated as duplicates under our guideline. In other words, even a human annotator with access only to VigiBase cannot achieve perfect recall against these reference sets. It also means that comparison to duplicate detection algorithms that leverage the richer information available in a national database is difficult, even when evaluating against the same reference set (eg, the FDA silver and gold datasets are also used in [11]). Since there is nothing specializing vigiMatch2025 to global databases, we believe the performance of vigiMatch2025 would be comparable with that observed here, if deployed on smaller or more localized databases. Indeed, in such settings, including the additional available information as features to the model would likely improve performance. This could also facilitate duplicate detection at the time of data entry or report submission, which begs further development and testing in future studies.

The externally indicated feature is a strong predictor in vigiMatch2025, and by construction it was highly prevalent in the test sets from Lareb, AEMPS, and MHRA, while the precision evaluations highlighted that such a link is relatively rare in general. Indeed, there are only 65,000 pairs in VigiBase that can be linked in this way. However, due to the substantial improvement in performance where the feature was applicable and the absence of significant performance reduction elsewhere, we decided to retain it. Indeed, the increased performance on the FDA datasets, where there is minimal presence of this feature, is encouraging that performance generalizes.

Areas for future research might include investigation of more advanced blocking schemes, to further improve computational efficiency. Additional features such as drug dosages may also be explored. Their low prevalence makes them difficult to incorporate effectively in a generalist model; however, the human annotators acknowledged their value as evidence for or against duplication. There is additionally some scope to improve the representation of report dates in the model. While the date embedding vectors successfully capture a more comprehensive and nuanced picture of a report’s timeline, they do not penalize mismatches more than missing dates, nor account for the number of dates matching on a report. The hit-miss model for onset date was retained in the model to partially mitigate this shortcoming; however, it would be desirable in future models to capture date information within one feature. Further research is also needed to better characterize performance across data subsets, which could help identify characteristics relevant to detecting data drift. For deployment, tolerance intervals for model drifts should also be determined.

In our annotation, we used more granular labels, also noting whether the reports in a pair were likely otherwise related. That many of the false positives in the precision evaluation were otherwise related suggests that the mistakes the model makes are generally understandable. However, this also raises the possibility of further refining the model with a second classifier to distinguish between otherwise related and duplicate reports. Such a model would not be constrained to be so computationally inexpensive, and so more sophisticated models, such as large language models, could be considered.

### Conclusions

Our study introduces a new predictive model, vigiMatch2025, for duplicate detection in databases of adverse event reports. We demonstrate that the model outperforms the previous state-of-the-art model, vigiMatch2017, in every way that we measured, while retaining desirable properties such as low computational cost and explainability.

## Appendix

Multimedia Appendix 1 Supplementary materials supporting the study, including an annotation guideline and structured case level analysis.

## Abbreviations

AEMPS: Agencia Española de Medicamentos y Productos Sanitarios
ATC: Anatomical Therapeutic Chemical
CDER: Center for Drug Evaluation and Research
CREMLS: Consolidated Reporting Guidelines for Prognostic and Diagnostic Machine Learning Models
FAERS: FDA adverse event reporting system
FDA: US Food and Drug Administration
ICH: International Council for Harmonisation of Technical Requirements for Pharmaceuticals for Human Use
MedDRA: Medical Dictionary for Regulatory Activities terminology
MHRA: Medicines and Healthcare products Regulatory Agency
SVM: support vector machine
UMC: Uppsala Monitoring Centre
WHO: World Health Organization

## Data Availability

The data that support the findings of this study are not publicly available. Access to the data is restricted based on the conditions for access within the WHO Programme for International Drug Monitoring. Subject to these conditions, data are available from the authors on reasonable request.
