# Supplementary material for: A Scalable Predictive Modeling Approach to Duplicate Detection in Pharmacovigilance: Algorithm Development and Validation Study
Source: JMIR AI. 2026 Oct 9;5:e95282. doi: 10.2196/95282 (PMC13718098; doi:10.2196/95282)
Supplement: Supplementary file 1 [file ai_v5i1e95282_app1.docx]

## S1: CREMLS checklist

| **#** | **Item** | **Y** | **N** | **NA** | **Location / Reasoning** |
| --- | --- | --- | --- | --- | --- |
| **Study Details** | | | | | |
| 1.1 | *The medical/clinical task of interest* | Y |  |  | Section 1 |
| 1.2 | *The research question* | Y |  |  | Section 1 |
| 1.3 | *Current medical/clinical practice* | Y |  |  | Section 1 |
| 1.4 | *The known predictors and confounders to what is being predicted / diagnosed* | Y |  |  | Section 3.3.1 |
| 1.5 | *The overall study design* | Y |  |  | 3.4 |
| 1.6 | *The medical institutional setting(s)* | Y |  |  | 3.1 |
| 1.7 | *The target patient population* |  |  | NA | There is no target patient population as the model operates at the level of adverse event report pairs, as described in Section 3.1 |
| 1.8 | *The intended use of the ML model* | Y |  |  | Section 1 |
| 1.9 | *Existing model performance benchmarks for this task* | Y |  |  | Sections 4.2, 4.3, 4.4 + Supplementary S3 |
| 1.10 | *Ethical and other regulatory approvals obtained* |  |  | NA | Not applicable as study did not use human subjects or personal data. See Declarations. |
| **The Data** | | | | | |
| 2.1 | *Inclusion / exclusion criteria for the patient cohort* | Y |  |  | Section 3.1 |
| 2.2 | *Methods of data collection* |  |  | NA | The study did not involve data collection but relies on secondary use of existing data |
| 2.3 | *Bias introduced due to the method of data collection used* |  |  | NA | The study did not involve data collection but relies on secondary use of existing data |
| 2.4 | *Data characteristics* | Y |  |  | Section 3.1 (via citation) |
| 2.5 | *Methods of data transformations and preprocessing applied* | Y |  |  | Sections 3.3.3, 3.3.4, 3.3.5, 3.3.6 |
| 2.6 | *Known quality issues with the data* | Y |  |  | Section 1 |
| 2.7 | *Sample size calculation* |  | N |  | Size of recall sets depended on available data and size of model-specific precision tests were determined by expert availability rather than formal sample size calculations |
| 2.8 | *Data Availability* | Y |  |  | Declarations |
| **Methodology** | | | | | |
| 3.1 | *Strategies for handling missing data* | Y |  |  | Sections 3.3.3 and 3.3.5 |
| 3.2 | *Strategies for addressing class imbalance* |  |  |  | Sections 3.2 and 3.3.1 |
| 3.3 | *Strategies for reducing dimensionality of data* |  |  | NA | No need for dimension reduction identified in this study |
| 3.4 | *Strategies for handling outliers* |  |  | NA | Duplicate detection can be seen as an outlier detection problem in its own right |
| 3.5 | *Strategies for data augmentation* |  |  | NA | No need for data augmentation identified in this study |
| 3.6 | *Strategies for model pre-training* |  |  | NA | Models trained from scratch – no transfer learning involved |
| 3.7 | *The rationale for selecting the machine learning algorithm* | Y |  |  | Section 3.3.1 |
| 3.8 | *The method of evaluating model performance during training* | Y |  |  | Section 3.2.2 |
| 3.9 | *The method used for hyperparameter tuning* |  |  | NA | Very few hyperparameters involved and kept fixed (see Section 3.3.2) |
| 3.10 | *Model’s output adjustments* |  |  | NA | No model output adjustments performed |
| **Evaluation** | | | | | |
| 4.1 | *Performance metrics used to evaluate the model* | Y |  |  | Sections 3.4.1 and 3.4.2 |
| 4.2 | *The cost or consequence of errors* | Y |  |  | Section 1 |
| 4.3 | *The results of internal validation* | Y |  |  | Sections 4.2, 4.3 (internal test set ‘UMC’) and 4.4; performance metrics on training data not meaningful here |
| 4.4 | *The final model hyperparameters* | Y |  |  | Section 3.3.2 |
| 4.5 | *Model evaluation on an external dataset* | Y |  |  | Section 4.3 |
| 4.6 | *Characteristics relevant for detecting data shift and drift* |  | N |  | This is important, and a subject of future research. See discussion regarding this in Section 5. |
| **Explainability and Transparency** | | | | | |
| 5.1 | *The most important features and how they relate to the outcome(s)* | Y |  |  | Section 4.1 |
| 5.2 | *Plausibility of model outputs* | Y |  |  | Section 4.4 + Supplementary S3 |
| 5.3 | *Interpretation of model's results by an end-user* | Y |  |  | Section 5 |

## S2: Annotation Guideline

**General principles**

Based on all information available in VigiBase for the two reports make your best judgment, as if you had to place a bet, on one of the three categories. In most cases, it will not be possible to know for sure (without going back to the national centre for additional information, which is out of scope here) if two reports are duplicates or non-duplicates.

**Considerations in assessing duplicates**

- Specific information in narratives can be very valuable both in providing evidence for and against two reports being duplicates, though beware that otherwise related reports can have near-identical narratives as well
- Independent reports are unlikely to match on exact dates – especially so, when there are multiple matching dates linked to the same event – for example an adverse event onset or a drug start
- Matches on multiple drugs and adverse events can be unlikely for independent reports, especially if they are not a common combination of drugs and/or adverse events
- For decision support, you could consider how common a specific combination of drugs and/or adverse events is in VigiBase via Vigilyze’s search filter (remember the ‘and’ connection)
- If there is matching information of a general nature such as the drugs and adverse events or specific numerical features from the narrative but demographic information such as patient age and sex differs, reports may be more likely to be otherwise related than duplicates (e.g. same reporter, associated with a litigation case, reports of separate adverse events in the same patient, …)

**Categories**

- Non-duplicates
- Pairs of reports that should not be linked at all
- Flag - possible duplicates
- Reports that you would like to flag to an assessor as possible/likely duplicates for their consideration in reviewing a case series
- Reports that you would be fine with feeding back to a member organisation as suspected duplicates
- Reports that you would like to have the option to exclude in statistical signal detection
- Flag - Otherwise related reports
- Reports that are not independent of one another, but also not duplicates
- Reports for the same patient but different (occurrences of) adverse events
- Reports from the same primary reporter
- Reports for members of the same family
- Reports from the same study / article
- Reports related to the same litigation
- Reports associated with e.g. the same product quality, contamination, cold chain issue etc
- Reports of adverse event happening at the same mass administration event (e.g. the vaccination of the same class of school children),
- … but not reports just generally associated with the same mass administration campaign

## S3: Externally Linked Country Strings

When linking pairs via E2Br2 field A.1.11.2, only identifiers starting with one of the following strings were considered

 'DE-', 'GB-', 'FR-', 'IT-', 'NL-', 'GR-', 'BE-', 'PT-', 'CA-', 'ES-', 'PL-', 'CZ-', 'JP-', 'US-', 'DK-', 'FI-', 'AT-', 'AU-', 'SE-', 'PHH', 'RO-', 'IE-', 'CH-', 'HU-', 'NO-', 'IN-', 'SK-', 'BR-', 'HR-', 'EG-'

## S4: Illustrative Examples of Performance

### Drugs - True Positive

The following example is of a pair of reports which are a true duplicate pair identified by vigiMatch2025 but not by vigiMatch2017. The reports both describe the outcome of a patient involved in an study on platelet aggregation inhibition by Eptifibatide or Tirofiban during primary percutaneous interventions (1). The reports contain relatively little structured information about the patient, however it is clear from the narrative that the reports are describing the same fatal case from the study. Crucially, vigiMatch2025 is able to make use of the dates described in the narrative, as well as the fact that the reports were linked in the e2b field A.1.11.2 as being duplicates, both of which vigiMatch2017 does not have access to, to add to the evidence from the matching drug and reaction and correctly identify the pair as a duplicates.

|  | Report 1 | Report 2 | vigiMatch2025 Score |
| --- | --- | --- | --- |
| Sex | Not reported | Not reported | 0 |
| Age | Not reported | Not reported | 0 |
| Dates | 2 matching and one mismatching date in the narrative  No reaction dates reported | | 0.59  0 |
| Drugs/Reactions | 1 matching reaction  1 matching drug | | 2.29 |
| Externally Linked | Yes | | 1.76 |
| Total Score |  |  | 4.63 |
| Patient Heuristic | PASS | | 0.59 |

### Drugs - False Negative

The following example is a of a pair of reports which are a true duplicate pair identified by vigiMatch 2017 but not by vigiMatch2025. The reports both come from a high population country in Europe with many reports in VigiBase. The age appears to have been miscoded in one of the reports, since the patient is described in both free text narratives as being 48, leading to a significant negative contribution to the vigiMatch score. This leads to the pair failing to pass the patient heuristic, and thus being classified as non duplicates, The base model would have otherwise classified them as a duplicate. It is possible that one describes the age at onset of the first reaction, and the other the age at the time of reporting, since the narratives describe a clinical course spanning several years. Moreover, whilst both narratives describe, in detail, the same clinical course, since one report seems to be a follow-up of the other, there are several dates, mostly relating to laboratory tests, in one narrative which don’t appear in the other, leading to a relatively low score from the dates, despite many matches in the reaction and drug fields.

|  | Report 1 | Report 2 | vigiMatch2024 Score |
| --- | --- | --- | --- |
| Sex | Male | Male | 0.15 |
| Age | 42-43 years | 48-49 years | -0.46 |
| Dates |  | | 0.2  1.04 |
| Drugs/Reactions | 3 matching reactions  1 mismatching but semantically similar reaction on each report  5 distinct matching drugs | | 4.22 |
| Externally Linked | Yes | | 1.76 |
| Total Score |  |  | 6.93 |
| Patient Heuristic | FAIL | | -0.1 |

### Drugs - False Positive

The following example is a pair of reports which are not considered a duplicate pair under our annotation guideline, but which vigiMatch2025 identifies as a suspected duplicate. Both reports come from a large population country in the Americas with many reports in VigiBase. These cases are practically identical, and very well might be related, however do not contain enough evidence to label them as such according to our annotation guideline.

|  | Report 1 | Report 2 | vigiMatch2025 Score |
| --- | --- | --- | --- |
| Sex | Male | Male | 0.15 |
| Age | Not reported | Not reported | 0 |
| Dates | No reported dates | | 0  0 |
| Drugs/Reactions | 2 matching reactions  3 matching drugs | | 2.78 |
| Externally Linked | No | | 0 |
| Total Score |  |  | 2.93 |
| Patient Heuristic | PASS | | 0.15 |

### Drugs - True Negative

This example is a pair of reports correctly identified as non duplicates by vigiMatch2025, and spuriously identified as duplicates by vigiMatch2017. The reports come from a high population country in Africa with relatively few reports in VigiBase. The reports describe two patients who both experienced the same reaction within a day of one another, as a suspected result of taking the same three medications. Since the country has few reports, and all of the drugs and reactions are globally uncommon, vigiMatch2017 therefore spuriously identified these as duplicates. However, in the context of the country, the drugs and reactions are in fact rather common. The reaction occurs on around 1/6 cases from this country, and 1/4 cases have at least one of the drugs. Moreover, the patients have different reported sex. Whilst not used in either model, the patients are also reported to have substantially different body weights. This pair doesn’t make it past the patient heuristic, although it’s key to note that the pair wouldn’t have been considered a duplicate even if the sexes had matched.

|  | Report 1 | Report 2 | vigiMatch2025 Score |
| --- | --- | --- | --- |
| Sex | Male | Female | -0.53 |
| Age | Not reported | 23-24 years | 0 |
| Dates | 2 matching distinct dates  Earliest reaction date mismatch by 1 day | | 0.25  0.25 |
| Drugs/Reactions | 2 matching reactions  3 matching drugs | | 0.36 |
| Externally Linked | No | | 0 |
| Total Score |  |  | 0.33 |
| Patient Heuristic | FAIL | | -0.28 |

### Vaccines - False Positive

These two reports are identified as a suspected duplicates by vigiMatch2025, but are labelled as non duplicates according to this paper’s annotation guideline. The reports both come from a high population country in the Americas with many reports in VigiBase. These reports contain an unusually large number of reported reactions, the vast majority of which are representing diagnostic tests and their results (which are rare to be coded as reactions in VigiBase). The age hit-miss model also doesn’t take into account the magnitude of the patient age when determining the match score, where a mismatch of a few months should probably be much more significant in infants, like this case, than in adults. Here the ages are rewarded for being close.

|  | Report 1 | Report 2 | vigiMatch2025Score |
| --- | --- | --- | --- |
| Sex | Female | Female | 0.29 |
| Age | 109-146 days | 36-73 days | 0.3 |
| Dates | 2 distinct dates on each report, none of which match  Earliest onset dates differ by 18 months | | 0  -0.93 |
| Drugs/Reactions | 29 matching reactions  86 mismatching reactions  4 matching vaccines | | 5.84 |
| Externally Linked | No | | 0 |
| Total Score |  |  | 3.14 |
| Patient Heuristic | PASS | | 0.59 |

### Vaccines - False Negative

These two reports are labelled as a suspected duplicate pair by the human annotator, but vigiMatch2025 fails to identify them as such. The reports come from a medium sized European country with many reports in VigiBase. The pair appears to be a report from the first dose of a childhood schedule of vaccines, and the other report referring to the same adverse event but including information up until the third dose, so a possible follow-up. This pair exemplifies the high standard of evidence necessary for a vaccine pair to be suspected as a duplicate according to our annotation guideline, but it may also reflect a computational deviation. Whilst many of the drugs and reactions are matching, they have a high degree of correlation for this country. Before it is moderated by the SVM coefficient, the drug/reaction binary hitmiss model for this pair comprises;

- + 7.36 for the drug model
- + 9.95 for the reaction model
- - 22.19 for the correlation compensation

Leading to an overall negative contribution to the score. This behaviour is incorrect (the resulting score for a set of matching drugs and adverse events should always be positive), and arises from an edge case where the correlations between drugs and adverse events are extremely high and the drugs/adverse reactions make up a significant proportion of that country’s dataset, leading to a breakdown in one of the approximations used in the computation of the correlation compensation.(2)

|  | Report 1 | Report 2 | vigiMatch2025 Score |
| --- | --- | --- | --- |
| Sex | Female | Female | 0.29 |
| Age | Not reported | 1-2 years | 0 |
| Dates | 3 matching dates out of 5 total distinct dates  Earliest onset date matches | | 0.39  1.26 |
| Drugs/Reactions | 8 matching reactions  3 mismatching reactions  3 matching vaccines/drugs | | -0.67 |
| Externally Linked | Yes | | 2.98 |
| Total Score |  |  | 4.26 |
| Patient Heuristic | PASS | | 0.68 |
